# Supplementary material for: Modeling glioblastoma heterogeneity as a dynamic network of cell states
Source: Mol Syst Biol. 2021 Sep 16;17(9):e10105. doi: 10.15252/msb.202010105 (PMC8444284; doi:10.15252/msb.202010105)
Supplement: Supplementary file 6 — Source Data for Figure 5 [file MSB-17-e10105-s004.zip › Figure5A_sourcedata/GSEA_3071/nosigdb_stateII.GseaPreranked.1622795151171/GAROFANO_IAVARONE_PPR_SUBTYPE.html]

Details for gene set GAROFANO\_IAVARONE\_PPR\_SUBTYPE[GSEA]

|  || Dataset | state23071 |
| Phenotype | NoPhenotypeAvailable |
| Upregulated in class | na\_pos |
| GeneSet | GAROFANO\_IAVARONE\_PPR\_SUBTYPE |
| Enrichment Score (ES) | 0.63703656 |
| Normalized Enrichment Score (NES) | 4.781505 |
| Nominal p-value | 0.0 |
| FDR q-value | 0.0 |
| FWER p-Value | 0.0 |
Table: GSEA Results Summary

  

Fig 1: Enrichment plot: GAROFANO\_IAVARONE\_PPR\_SUBTYPE      
 Profile of the Running ES Score & Positions of GeneSet Members on the Rank Ordered List

  

| PROBE | GENE SYMBOL | GENE\_TITLE | RANK IN GENE LIST | RANK METRIC SCORE | RUNNING ES | CORE ENRICHMENT || 1 | CLSPN |  |  | 1 | 1.984 | 0.0083 | Yes |
| 2 | HIST1H4C |  |  | 2 | 1.902 | 0.0164 | Yes |
| 3 | RRM2 |  |  | 3 | 1.857 | 0.0244 | Yes |
| 4 | HIST1H1B |  |  | 7 | 1.669 | 0.0308 | Yes |
| 5 | TYMS |  |  | 8 | 1.665 | 0.0380 | Yes |
| 6 | MCM10 |  |  | 9 | 1.654 | 0.0451 | Yes |
| 7 | CDC6 |  |  | 11 | 1.631 | 0.0518 | Yes |
| 8 | CDK1 |  |  | 14 | 1.540 | 0.0579 | Yes |
| 9 | ZWINT |  |  | 16 | 1.510 | 0.0641 | Yes |
| 10 | HJURP |  |  | 17 | 1.490 | 0.0705 | Yes |
| 11 | ATAD5 |  |  | 18 | 1.469 | 0.0769 | Yes |
| 12 | FEN1 |  |  | 19 | 1.464 | 0.0831 | Yes |
| 13 | HIST2H2AC |  |  | 20 | 1.462 | 0.0894 | Yes |
| 14 | BRCA2 |  |  | 22 | 1.445 | 0.0954 | Yes |
| 15 | UBE2C |  |  | 23 | 1.438 | 0.1016 | Yes |
| 16 | RAD51AP1 |  |  | 25 | 1.384 | 0.1072 | Yes |
| 17 | CENPK |  |  | 26 | 1.382 | 0.1132 | Yes |
| 18 | MYBL2 |  |  | 28 | 1.362 | 0.1188 | Yes |
| 19 | FAM111B |  |  | 29 | 1.361 | 0.1246 | Yes |
| 20 | DTL |  |  | 33 | 1.330 | 0.1296 | Yes |
| 21 | RRM1 |  |  | 37 | 1.310 | 0.1344 | Yes |
| 22 | GINS2 |  |  | 40 | 1.306 | 0.1395 | Yes |
| 23 | CDCA5 |  |  | 41 | 1.289 | 0.1450 | Yes |
| 24 | TK1 |  |  | 42 | 1.281 | 0.1505 | Yes |
| 25 | AURKB |  |  | 44 | 1.276 | 0.1557 | Yes |
| 26 | MCM7 |  |  | 45 | 1.273 | 0.1612 | Yes |
| 27 | HELLS |  |  | 46 | 1.259 | 0.1666 | Yes |
| 28 | BRCA1 |  |  | 49 | 1.249 | 0.1715 | Yes |
| 29 | CDT1 |  |  | 51 | 1.244 | 0.1766 | Yes |
| 30 | GINS1 |  |  | 52 | 1.239 | 0.1819 | Yes |
| 31 | CCNA2 |  |  | 53 | 1.230 | 0.1872 | Yes |
| 32 | NEIL3 |  |  | 54 | 1.228 | 0.1925 | Yes |
| 33 | DNAJC9 |  |  | 56 | 1.217 | 0.1974 | Yes |
| 34 | SKA3 |  |  | 57 | 1.215 | 0.2026 | Yes |
| 35 | PCNA |  |  | 58 | 1.214 | 0.2079 | Yes |
| 36 | CDC45 |  |  | 59 | 1.212 | 0.2131 | Yes |
| 37 | MAD2L1 |  |  | 61 | 1.199 | 0.2180 | Yes |
| 38 | UBE2T |  |  | 62 | 1.192 | 0.2231 | Yes |
| 39 | ATAD2 |  |  | 63 | 1.189 | 0.2282 | Yes |
| 40 | PRIM1 |  |  | 65 | 1.170 | 0.2330 | Yes |
| 41 | MSH2 |  |  | 66 | 1.163 | 0.2380 | Yes |
| 42 | RFC3 |  |  | 67 | 1.157 | 0.2429 | Yes |
| 43 | SPC25 |  |  | 68 | 1.156 | 0.2479 | Yes |
| 44 | MELK |  |  | 69 | 1.145 | 0.2528 | Yes |
| 45 | POLD3 |  |  | 70 | 1.143 | 0.2577 | Yes |
| 46 | TOP2A |  |  | 72 | 1.132 | 0.2623 | Yes |
| 47 | KIF2C |  |  | 73 | 1.132 | 0.2672 | Yes |
| 48 | MCM5 |  |  | 74 | 1.128 | 0.2721 | Yes |
| 49 | DSN1 |  |  | 75 | 1.122 | 0.2769 | Yes |
| 50 | MCM4 |  |  | 81 | 1.110 | 0.2803 | Yes |
| 51 | RBL1 |  |  | 82 | 1.110 | 0.2851 | Yes |
| 52 | GMNN |  |  | 83 | 1.108 | 0.2899 | Yes |
| 53 | KIFC1 |  |  | 88 | 1.094 | 0.2935 | Yes |
| 54 | SKA1 |  |  | 91 | 1.078 | 0.2976 | Yes |
| 55 | XRCC2 |  |  | 92 | 1.076 | 0.3022 | Yes |
| 56 | NDC80 |  |  | 94 | 1.074 | 0.3066 | Yes |
| 57 | POLA2 |  |  | 95 | 1.068 | 0.3112 | Yes |
| 58 | MASTL |  |  | 96 | 1.063 | 0.3158 | Yes |
| 59 | TMPO |  |  | 99 | 1.056 | 0.3198 | Yes |
| 60 | NCAPG |  |  | 100 | 1.056 | 0.3243 | Yes |
| 61 | MND1 |  |  | 104 | 1.036 | 0.3280 | Yes |
| 62 | MKI67 |  |  | 107 | 1.031 | 0.3319 | Yes |
| 63 | STIL |  |  | 108 | 1.031 | 0.3363 | Yes |
| 64 | FANCA |  |  | 109 | 1.029 | 0.3407 | Yes |
| 65 | BRIP1 |  |  | 110 | 1.029 | 0.3451 | Yes |
| 66 | TPX2 |  |  | 111 | 1.025 | 0.3496 | Yes |
| 67 | RFC2 |  |  | 112 | 1.022 | 0.3539 | Yes |
| 68 | CDCA8 |  |  | 113 | 1.018 | 0.3583 | Yes |
| 69 | TRIP13 |  |  | 114 | 1.014 | 0.3627 | Yes |
| 70 | KIF11 |  |  | 115 | 1.014 | 0.3670 | Yes |
| 71 | NCAPH |  |  | 116 | 1.013 | 0.3714 | Yes |
| 72 | NCAPD3 |  |  | 117 | 1.013 | 0.3758 | Yes |
| 73 | DUT |  |  | 118 | 1.009 | 0.3801 | Yes |
| 74 | ASF1B |  |  | 120 | 1.007 | 0.3842 | Yes |
| 75 | SMC2 |  |  | 123 | 1.005 | 0.3879 | Yes |
| 76 | NUF2 |  |  | 125 | 1.004 | 0.3920 | Yes |
| 77 | CHAF1A |  |  | 127 | 0.999 | 0.3960 | Yes |
| 78 | CKS1B |  |  | 128 | 0.999 | 0.4003 | Yes |
| 79 | TACC3 |  |  | 130 | 0.992 | 0.4043 | Yes |
| 80 | FANCI |  |  | 138 | 0.984 | 0.4067 | Yes |
| 81 | WDR76 |  |  | 142 | 0.975 | 0.4101 | Yes |
| 82 | ESCO2 |  |  | 144 | 0.971 | 0.4140 | Yes |
| 83 | EXO1 |  |  | 145 | 0.969 | 0.4182 | Yes |
| 84 | CKAP2L |  |  | 148 | 0.959 | 0.4218 | Yes |
| 85 | RANBP1 |  |  | 149 | 0.958 | 0.4259 | Yes |
| 86 | FANCB |  |  | 151 | 0.956 | 0.4297 | Yes |
| 87 | FIGNL1 |  |  | 152 | 0.954 | 0.4338 | Yes |
| 88 | DSCC1 |  |  | 155 | 0.946 | 0.4374 | Yes |
| 89 | BLM |  |  | 156 | 0.942 | 0.4414 | Yes |
| 90 | NCAPG2 |  |  | 159 | 0.937 | 0.4449 | Yes |
| 91 | KIF23 |  |  | 161 | 0.936 | 0.4487 | Yes |
| 92 | RTKN2 |  |  | 166 | 0.931 | 0.4516 | Yes |
| 93 | GTSE1 |  |  | 167 | 0.929 | 0.4556 | Yes |
| 94 | CEP78 |  |  | 170 | 0.927 | 0.4591 | Yes |
| 95 | CDCA2 |  |  | 171 | 0.922 | 0.4630 | Yes |
| 96 | MCM6 |  |  | 176 | 0.906 | 0.4659 | Yes |
| 97 | CENPM |  |  | 178 | 0.901 | 0.4695 | Yes |
| 98 | PLK4 |  |  | 205 | 0.870 | 0.4664 | Yes |
| 99 | H2AFZ |  |  | 213 | 0.864 | 0.4682 | Yes |
| 100 | CEP152 |  |  | 217 | 0.860 | 0.4711 | Yes |
| 101 | CENPH |  |  | 221 | 0.855 | 0.4740 | Yes |
| 102 | BUB1B |  |  | 223 | 0.851 | 0.4774 | Yes |
| 103 | MRTO4 |  |  | 228 | 0.847 | 0.4800 | Yes |
| 104 | CHAF1B |  |  | 229 | 0.845 | 0.4836 | Yes |
| 105 | ASPM |  |  | 231 | 0.842 | 0.4870 | Yes |
| 106 | EZH2 |  |  | 233 | 0.839 | 0.4903 | Yes |
| 107 | DBF4 |  |  | 240 | 0.832 | 0.4923 | Yes |
| 108 | RACGAP1 |  |  | 241 | 0.831 | 0.4959 | Yes |
| 109 | PRC1 |  |  | 242 | 0.830 | 0.4995 | Yes |
| 110 | KNTC1 |  |  | 249 | 0.825 | 0.5014 | Yes |
| 111 | LMNB1 |  |  | 251 | 0.824 | 0.5047 | Yes |
| 112 | CHEK1 |  |  | 252 | 0.822 | 0.5082 | Yes |
| 113 | KIF18B |  |  | 254 | 0.815 | 0.5115 | Yes |
| 114 | DNA2 |  |  | 263 | 0.797 | 0.5128 | Yes |
| 115 | DNMT1 |  |  | 265 | 0.792 | 0.5159 | Yes |
| 116 | CENPI |  |  | 269 | 0.788 | 0.5185 | Yes |
| 117 | HMGB2 |  |  | 276 | 0.780 | 0.5203 | Yes |
| 118 | SMC4 |  |  | 279 | 0.777 | 0.5231 | Yes |
| 119 | CENPE |  |  | 284 | 0.775 | 0.5254 | Yes |
| 120 | RFWD3 |  |  | 290 | 0.769 | 0.5274 | Yes |
| 121 | BARD1 |  |  | 295 | 0.760 | 0.5296 | Yes |
| 122 | C1orf112 |  |  | 298 | 0.756 | 0.5323 | Yes |
| 123 | CENPA |  |  | 303 | 0.751 | 0.5345 | Yes |
| 124 | POLE |  |  | 305 | 0.750 | 0.5374 | Yes |
| 125 | CENPO |  |  | 307 | 0.747 | 0.5404 | Yes |
| 126 | KIF4A |  |  | 309 | 0.746 | 0.5433 | Yes |
| 127 | CENPN |  |  | 320 | 0.737 | 0.5438 | Yes |
| 128 | CCNF |  |  | 321 | 0.737 | 0.5470 | Yes |
| 129 | PKMYT1 |  |  | 322 | 0.736 | 0.5502 | Yes |
| 130 | CENPW |  |  | 330 | 0.727 | 0.5514 | Yes |
| 131 | SPC24 |  |  | 340 | 0.719 | 0.5522 | Yes |
| 132 | LIG1 |  |  | 341 | 0.718 | 0.5552 | Yes |
| 133 | ARHGAP11A |  |  | 346 | 0.712 | 0.5572 | Yes |
| 134 | LMNB2 |  |  | 352 | 0.709 | 0.5590 | Yes |
| 135 | DTYMK |  |  | 364 | 0.701 | 0.5591 | Yes |
| 136 | CENPJ |  |  | 376 | 0.695 | 0.5592 | Yes |
| 137 | KIF14 |  |  | 378 | 0.693 | 0.5619 | Yes |
| 138 | E2F8 |  |  | 379 | 0.691 | 0.5649 | Yes |
| 139 | KIF18A |  |  | 392 | 0.683 | 0.5646 | Yes |
| 140 | EXOSC9 |  |  | 399 | 0.679 | 0.5660 | Yes |
| 141 | TMEM97 |  |  | 403 | 0.676 | 0.5681 | Yes |
| 142 | KIF15 |  |  | 404 | 0.676 | 0.5710 | Yes |
| 143 | SAE1 |  |  | 418 | 0.669 | 0.5704 | Yes |
| 144 | KPNA2 |  |  | 420 | 0.669 | 0.5730 | Yes |
| 145 | FOXM1 |  |  | 423 | 0.667 | 0.5754 | Yes |
| 146 | SMC3 |  |  | 425 | 0.667 | 0.5780 | Yes |
| 147 | INCENP |  |  | 432 | 0.660 | 0.5792 | Yes |
| 148 | RMI1 |  |  | 433 | 0.660 | 0.5821 | Yes |
| 149 | HNRNPAB |  |  | 435 | 0.659 | 0.5846 | Yes |
| 150 | ZWILCH |  |  | 436 | 0.659 | 0.5875 | Yes |
| 151 | POLA1 |  |  | 443 | 0.654 | 0.5887 | Yes |
| 152 | PIF1 |  |  | 445 | 0.653 | 0.5912 | Yes |
| 153 | RAD54L |  |  | 456 | 0.648 | 0.5914 | Yes |
| 154 | WDR62 |  |  | 459 | 0.646 | 0.5936 | Yes |
| 155 | DBF4B |  |  | 473 | 0.640 | 0.5929 | Yes |
| 156 | DCTPP1 |  |  | 477 | 0.638 | 0.5949 | Yes |
| 157 | SSRP1 |  |  | 478 | 0.638 | 0.5976 | Yes |
| 158 | CKLF |  |  | 482 | 0.637 | 0.5996 | Yes |
| 159 | POP7 |  |  | 502 | 0.628 | 0.5973 | Yes |
| 160 | BIRC5 |  |  | 505 | 0.628 | 0.5994 | Yes |
| 161 | RAD51 |  |  | 506 | 0.627 | 0.6021 | Yes |
| 162 | EXOSC2 |  |  | 509 | 0.626 | 0.6043 | Yes |
| 163 | ECT2 |  |  | 510 | 0.626 | 0.6070 | Yes |
| 164 | GEN1 |  |  | 517 | 0.620 | 0.6081 | Yes |
| 165 | HAUS8 |  |  | 527 | 0.613 | 0.6083 | Yes |
| 166 | CDK2 |  |  | 530 | 0.612 | 0.6104 | Yes |
| 167 | C5orf34 |  |  | 533 | 0.611 | 0.6125 | Yes |
| 168 | RCC1 |  |  | 549 | 0.603 | 0.6111 | Yes |
| 169 | C18orf54 |  |  | 550 | 0.603 | 0.6137 | Yes |
| 170 | CDC25A |  |  | 572 | 0.589 | 0.6107 | Yes |
| 171 | CENPF |  |  | 575 | 0.588 | 0.6127 | Yes |
| 172 | TIMELESS |  |  | 591 | 0.583 | 0.6113 | Yes |
| 173 | DDX11 |  |  | 596 | 0.579 | 0.6127 | Yes |
| 174 | CEP135 |  |  | 598 | 0.578 | 0.6149 | Yes |
| 175 | EXOSC8 |  |  | 604 | 0.576 | 0.6161 | Yes |
| 176 | MSH6 |  |  | 611 | 0.574 | 0.6170 | Yes |
| 177 | SMC1A |  |  | 618 | 0.570 | 0.6178 | Yes |
| 178 | CKAP2 |  |  | 619 | 0.569 | 0.6203 | Yes |
| 179 | CDC25C |  |  | 626 | 0.568 | 0.6211 | Yes |
| 180 | SASS6 |  |  | 637 | 0.562 | 0.6209 | Yes |
| 181 | NCL |  |  | 638 | 0.560 | 0.6233 | Yes |
| 182 | SPAG5 |  |  | 642 | 0.555 | 0.6249 | Yes |
| 183 | HAUS1 |  |  | 649 | 0.552 | 0.6257 | Yes |
| 184 | NUSAP1 |  |  | 654 | 0.550 | 0.6270 | Yes |
| 185 | LSM6 |  |  | 657 | 0.550 | 0.6288 | Yes |
| 186 | H2AFX |  |  | 671 | 0.544 | 0.6277 | Yes |
| 187 | MNS1 |  |  | 672 | 0.543 | 0.6301 | Yes |
| 188 | NUP205 |  |  | 705 | 0.531 | 0.6239 | Yes |
| 189 | RIF1 |  |  | 713 | 0.529 | 0.6243 | Yes |
| 190 | TOE1 |  |  | 714 | 0.528 | 0.6266 | Yes |
| 191 | GPSM2 |  |  | 719 | 0.526 | 0.6278 | Yes |
| 192 | BAZ1B |  |  | 721 | 0.525 | 0.6298 | Yes |
| 193 | EME1 |  |  | 726 | 0.525 | 0.6310 | Yes |
| 194 | POLD1 |  |  | 729 | 0.523 | 0.6327 | Yes |
| 195 | MCM2 |  |  | 734 | 0.521 | 0.6339 | Yes |
| 196 | HAUS3 |  |  | 739 | 0.519 | 0.6351 | Yes |
| 197 | NASP |  |  | 741 | 0.518 | 0.6370 | Yes |
| 198 | POLQ |  |  | 771 | 0.508 | 0.6316 | No |
| 199 | DCLRE1C |  |  | 786 | 0.503 | 0.6300 | No |
| 200 | CCDC150 |  |  | 804 | 0.496 | 0.6277 | No |
| 201 | CPSF4 |  |  | 807 | 0.494 | 0.6293 | No |
| 202 | SUV39H2 |  |  | 821 | 0.491 | 0.6279 | No |
| 203 | LIN54 |  |  | 823 | 0.490 | 0.6298 | No |
| 204 | TUBA1B |  |  | 862 | 0.481 | 0.6218 | No |
| 205 | CASP2 |  |  | 884 | 0.474 | 0.6183 | No |
| 206 | CCDC138 |  |  | 903 | 0.470 | 0.6156 | No |
| 207 | DEPDC1B |  |  | 906 | 0.470 | 0.6171 | No |
| 208 | ZNF90 |  |  | 921 | 0.466 | 0.6154 | No |
| 209 | SUV39H1 |  |  | 925 | 0.464 | 0.6166 | No |
| 210 | HIRIP3 |  |  | 936 | 0.462 | 0.6159 | No |
| 211 | LIN9 |  |  | 949 | 0.459 | 0.6147 | No |
| 212 | PTBP1 |  |  | 970 | 0.452 | 0.6114 | No |
| 213 | KIF22 |  |  | 975 | 0.450 | 0.6123 | No |
| 214 | FANCD2 |  |  | 976 | 0.450 | 0.6142 | No |
| 215 | TRIM28 |  |  | 1004 | 0.444 | 0.6090 | No |
| 216 | PLK1 |  |  | 1009 | 0.442 | 0.6098 | No |
| 217 | NR2C2AP |  |  | 1013 | 0.440 | 0.6109 | No |
| 218 | CASP8AP2 |  |  | 1025 | 0.436 | 0.6099 | No |
| 219 | ZNF519 |  |  | 1027 | 0.436 | 0.6115 | No |
| 220 | CHEK2 |  |  | 1073 | 0.424 | 0.6014 | No |
| 221 | POC1A |  |  | 1080 | 0.421 | 0.6017 | No |
| 222 | MYLK2 |  |  | 1092 | 0.419 | 0.6006 | No |
| 223 | CTDSPL2 |  |  | 1109 | 0.415 | 0.5981 | No |
| 224 | SRRT |  |  | 1121 | 0.414 | 0.5970 | No |
| 225 | DGCR8 |  |  | 1122 | 0.414 | 0.5988 | No |
| 226 | CBX5 |  |  | 1132 | 0.412 | 0.5982 | No |
| 227 | BUB1 |  |  | 1145 | 0.408 | 0.5968 | No |
| 228 | DPF1 |  |  | 1169 | 0.403 | 0.5924 | No |
| 229 | HNRNPD |  |  | 1177 | 0.401 | 0.5923 | No |
| 230 | FANCC |  |  | 1186 | 0.399 | 0.5919 | No |
| 231 | SNRPA |  |  | 1213 | 0.394 | 0.5867 | No |
| 232 | PTMA |  |  | 1233 | 0.390 | 0.5834 | No |
| 233 | STMN1 |  |  | 1249 | 0.387 | 0.5811 | No |
| 234 | ESPL1 |  |  | 1255 | 0.386 | 0.5814 | No |
| 235 | ZNF93 |  |  | 1265 | 0.384 | 0.5807 | No |
| 236 | BUD13 |  |  | 1283 | 0.381 | 0.5778 | No |
| 237 | RECQL4 |  |  | 1285 | 0.380 | 0.5792 | No |
| 238 | MTBP |  |  | 1318 | 0.375 | 0.5724 | No |
| 239 | RAD54B |  |  | 1327 | 0.373 | 0.5719 | No |
| 240 | ANP32E |  |  | 1334 | 0.371 | 0.5719 | No |
| 241 | RNASEH2A |  |  | 1341 | 0.369 | 0.5719 | No |
| 242 | ZNF829 |  |  | 1350 | 0.367 | 0.5713 | No |
| 243 | KLHL23 |  |  | 1369 | 0.362 | 0.5681 | No |
| 244 | ZNF85 |  |  | 1380 | 0.360 | 0.5671 | No |
| 245 | SENP1 |  |  | 1408 | 0.356 | 0.5615 | No |
| 246 | SPATA5 |  |  | 1424 | 0.352 | 0.5590 | No |
| 247 | TRAIP |  |  | 1430 | 0.351 | 0.5592 | No |
| 248 | PARP1 |  |  | 1431 | 0.351 | 0.5607 | No |
| 249 | LSM11 |  |  | 1438 | 0.350 | 0.5606 | No |
| 250 | PASK |  |  | 1450 | 0.347 | 0.5592 | No |
| 251 | RBM15 |  |  | 1467 | 0.345 | 0.5565 | No |
| 252 | GALE |  |  | 1476 | 0.344 | 0.5558 | No |
| 253 | DUS3L |  |  | 1543 | 0.332 | 0.5398 | No |
| 254 | CENPP |  |  | 1569 | 0.329 | 0.5347 | No |
| 255 | FUS |  |  | 1571 | 0.329 | 0.5358 | No |
| 256 | SCLY |  |  | 1592 | 0.325 | 0.5319 | No |
| 257 | ING2 |  |  | 1630 | 0.319 | 0.5235 | No |
| 258 | PXMP2 |  |  | 1665 | 0.315 | 0.5159 | No |
| 259 | PAXIP1 |  |  | 1673 | 0.313 | 0.5154 | No |
| 260 | LSM4 |  |  | 1676 | 0.313 | 0.5162 | No |
| 261 | GINS3 |  |  | 1694 | 0.312 | 0.5131 | No |
| 262 | PPP5C |  |  | 1704 | 0.310 | 0.5120 | No |
| 263 | PKNOX1 |  |  | 1711 | 0.309 | 0.5118 | No |
| 264 | HMGB1 |  |  | 1720 | 0.308 | 0.5110 | No |
| 265 | OIP5 |  |  | 1789 | 0.299 | 0.4943 | No |
| 266 | ZNF593 |  |  | 1804 | 0.298 | 0.4919 | No |
| 267 | C17orf53 |  |  | 1815 | 0.297 | 0.4905 | No |
| 268 | RAB8A |  |  | 1857 | 0.291 | 0.4810 | No |
| 269 | TAF5 |  |  | 1869 | 0.290 | 0.4793 | No |
| 270 | DNMT3B |  |  | 1881 | 0.288 | 0.4776 | No |
| 271 | THAP10 |  |  | 1935 | 0.281 | 0.4648 | No |
| 272 | DHX15 |  |  | 1985 | 0.275 | 0.4531 | No |
| 273 | DCLRE1A |  |  | 2007 | 0.272 | 0.4487 | No |
| 274 | ZIK1 |  |  | 2021 | 0.270 | 0.4464 | No |
| 275 | ERI2 |  |  | 2034 | 0.269 | 0.4444 | No |
| 276 | SKA2 |  |  | 2061 | 0.266 | 0.4387 | No |
| 277 | PPIA |  |  | 2072 | 0.265 | 0.4372 | No |
| 278 | ATRIP |  |  | 2075 | 0.265 | 0.4378 | No |
| 279 | TMEM39B |  |  | 2090 | 0.263 | 0.4352 | No |
| 280 | RBM14 |  |  | 2103 | 0.262 | 0.4332 | No |
| 281 | CKAP5 |  |  | 2135 | 0.258 | 0.4261 | No |
| 282 | CEP72 |  |  | 2137 | 0.258 | 0.4270 | No |
| 283 | CALML4 |  |  | 2161 | 0.256 | 0.4220 | No |
| 284 | SEPHS1 |  |  | 2188 | 0.253 | 0.4162 | No |
| 285 | ATP9B |  |  | 2276 | -0.256 | 0.3943 | No |
| 286 | CYTH2 |  |  | 2531 | -0.291 | 0.3285 | No |
| 287 | RSBN1L |  |  | 2546 | -0.293 | 0.3261 | No |
| 288 | PLEKHA8 |  |  | 2614 | -0.303 | 0.3097 | No |
| 289 | SECISBP2 |  |  | 2802 | -0.335 | 0.2618 | No |
| 290 | ZNF664 |  |  | 2824 | -0.340 | 0.2577 | No |
| 291 | ZNF43 |  |  | 2872 | -0.348 | 0.2468 | No |
| 292 | GTF2IRD2 |  |  | 2946 | -0.364 | 0.2290 | No |
| 293 | TIA1 |  |  | 2978 | -0.369 | 0.2224 | No |
| 294 | XPA |  |  | 3062 | -0.388 | 0.2022 | No |
| 295 | ZNF117 |  |  | 3117 | -0.403 | 0.1897 | No |
| 296 | CREBBP |  |  | 3329 | -0.466 | 0.1360 | No |
| 297 | ADD2 |  |  | 3492 | -0.538 | 0.0955 | No |
| 298 | GAB1 |  |  | 3684 | -0.670 | 0.0479 | No |
| 299 | LCORL |  |  | 3718 | -0.698 | 0.0422 | No |
| 300 | ZNF704 |  |  | 3750 | -0.729 | 0.0372 | No |
| 301 | TOP2B |  |  | 3777 | -0.767 | 0.0336 | No |
| 302 | SALL3 |  |  | 3778 | -0.768 | 0.0369 | No |
| 303 | MAML2 |  |  | 3793 | -0.783 | 0.0366 | No |
| 304 | CDKN1B |  |  | 3812 | -0.800 | 0.0353 | No |
| 305 | IDH1 |  |  | 3842 | -0.832 | 0.0312 | No |
| 306 | CCDC88A |  |  | 3854 | -0.848 | 0.0319 | No |
| 307 | MEX3A |  |  | 3866 | -0.867 | 0.0327 | No |
| 308 | TRIB2 |  |  | 3944 | -1.041 | 0.0169 | No |
| 309 | SOX11 |  |  | 3977 | -1.147 | 0.0134 | No |
| 310 | PGAP1 |  |  | 3981 | -1.164 | 0.0176 | No |
| 311 | TUBA1A |  |  | 4006 | -1.302 | 0.0168 | No |
| 312 | MAP2 |  |  | 4051 | -1.673 | 0.0124 | No |
Table: GSEA details [plain text format]

  

Fig 2: GAROFANO\_IAVARONE\_PPR\_SUBTYPE: Random ES distribution      
 Gene set null distribution of ES for **GAROFANO\_IAVARONE\_PPR\_SUBTYPE**
